# Near-infrared imaging for automated tsetse pupae sex sorting in support of the sterile insect technique[Fn FN1]

**DOI:** 10.1051/parasite/2023019

**Published:** 2023-05-17

**Authors:** Rafael Argilés-Herrero, Gustavo Salvador-Herranz, Andrew G. Parker, Mario Zacarés, Assane G. Fall, Adji M. Gaye, Arooj Nawaz, Peter Takáč, Marc J.B. Vreysen, Chantel J. de Beer

**Affiliations:** 1 Joint FAO/IAEA Centre of Nuclear Techniques in Food and Agriculture, Insect Pest Control Laboratory, Department of Nuclear Sciences and Applications, International Atomic Energy Agency 1400 Vienna Austria; 2 Instituto Universitario de Investigación en Tecnología Centrada en el Ser Humano. Universitat Politècnica de València, Camino de Vera, s/n 46022 València Spain; 3 Roppersbergweg 15 2381 Laab im Walde Austria; 4 Departamento de Ciencias Básicas y Transversales. Facultad de Veterinaria y Ciencias Experimentales, Universidad Católica de Valencia San Vicente Mártir C/Guillem de Castro 94 46001 Valencia Spain; 5 Institut Sénégalais de Recherches Agricoles, Laboratoire National de l’Élevage et de Recherches Vétérinaires, Service de Bio-écologie et Pathologies Parasitaires BP 2057 Hann, Dakar Senegal; 6 Ministère de l’Élevage et des Productions animales, Sphères Ministérielles de Diamniadio BP 13 000 Dakar Senegal; 7 Institute of Zoology, Slovak Academy of Sciences Dúbravská cesta 9 845 06 Bratislava Slovakia; 8 Scientica, Ltd. Hybešova 33 831 06 Bratislava Slovakia

**Keywords:** Glossina, Sterile insect technique (SIT), Genetic management of vectors, Sex separation, sexual dimorphism, NIR spectrum

## Abstract

Tsetse flies are the cyclical vectors of African trypanosomes and one of several methods to manage this vector is the sterile insect technique (SIT). The ability to determine the sex of tsetse pupae with the objective to separate the sexes before adult emergence has been a major goal for decades for tsetse management programmes with an SIT component. Tsetse females develop faster and pharate females inside the pupae melanise 1–2 days before males. This earlier melanisation can be detected by infrared cameras through the pupal shell, and the newly developed Near InfraRed Pupae Sex Sorter (NIRPSS) takes advantage of this. The melanisation process is not homogeneous for all fly organs and the pupa needs to be examined ventrally, dorsally and laterally to ensure accurate classification by an image analysis algorithm. When the pupae are maturing at a constant temperature of 24 °C and sorted at the appropriate age, 24 days post-larviposition for *Glossina palpalis gambiensis*, the sorting machine can efficiently separate the sexes. The recovered male pupae can then be sterilised for field releases of males, while the rest of the pupae can be used to maintain the laboratory colony. The sorting process with the new NIRPSS had no negative impact on adult emergence and flight ability. A mean male recovery of 62.82 ± 3.61% was enough to provide sterile males to an operational SIT programme, while mean contamination with females (4.69 ± 3.02%) was low enough to have no impact on the maintenance of a laboratory colony.

## Abstract (fr)

Les glossines sont les vecteurs cycliques des trypanosomes africains et la technique de l’insecte stérile (TIS) est l’une des méthodes de gestion de ce vecteur. La capacité à déterminer le sexe des pupes de glossines dans le but de séparer les sexes avant l’émergence des adultes a été un objectif majeur, pendant des décennies, pour les programmes de lutte contre les glossines avec une composante TIS. Les femelles tsé-tsé se développent plus rapidement et les pharates femelles à l’intérieur des pupes se mélanisent 1 à 2 jours avant les mâles. Cette mélanisation précoce peut être détectée par des caméras infrarouges à travers la coque de la pupe, ce que le nouveau trieur de sexe des pupes dans le proche infrarouge (TSPPIR) utilise. Le processus de mélanisation n’est pas homogène pour tous les organes de la mouche et la pupe doit être examinée ventralement, dorsalement et latéralement pour assurer une classification précise par un algorithme d’analyse d’image. Lorsque les pupes mûrissent à une température constante de 24 °C et sont triées à l’âge approprié, 24 jours après la larviposition pour *Glossina palpalis gambiensis*, la machine de tri peut séparer efficacement les sexes. Les pupes mâles récupérées peuvent ensuite être stérilisées pour les lâchers de mâles sur le terrain tandis que le reste des pupes peut être utilisé pour maintenir la colonie de laboratoire. Le processus de tri avec le nouveau TSPPIR n’a eu aucun impact négatif sur l’émergence et la capacité de vol des adultes. Une récupération moyenne des mâles de 62,82 ± 3,61% était suffisante pour fournir des mâles stériles à un programme TIS opérationnel, tandis que la contamination moyenne par les femelles (4,69 ± 3,02%) était suffisamment faible pour n’avoir aucun impact sur le maintien d’une colonie de laboratoire.

## Introduction

Tsetse flies (Diptera: Glossinidae) are the cyclical vectors of the trypanosomes that cause human African trypanosomosis (HAT) or sleeping sickness in humans, and African animal trypanosomosis (AAT) or nagana in livestock [22]. Human sleeping sickness has declined dramatically since the year 2000 mainly through case identification and treatment, and more recently, through localised vector management in transmission areas [34]. Nagana, however, remains widespread and is a major constraint to livestock production with estimated annual losses of $4.5 billion in sub-Saharan Africa [8]. Although anti-trypanocidal drugs can be used to treat animals, their efficacy is low in many areas due to the development of resistance of the parasites to the drugs. In the absence of a vaccine, vector management remains indispensable for Nagana management.

The sterile insect technique (SIT) [13] is one of several methods of tsetse management and is applied as a component of area-wide integrated pest management (AW-IPM) approaches. The SIT is based on the production of large numbers of insects of the target population, the sterilisation of the male sex using ionising radiation, and the sequential release of these sterile male insects in the target area where they compete with wild males for mating with wild females. This induces sterility in the native female population which results in a decline in the numbers of offspring with each generation and this can eventually lead to local eradication of the pest population.

In most insect species, it has not previously been possible to separate males from females in the larval or pupal stages. If pupae are irradiated, both sexes are sterilised and released and this reduces the efficiency of the SIT as the sterile males will likely mate preferentially with the sterile females released with them rather than seeking wild females. This results in a need to release three to four times more insects to achieve the desired level of induced sterility, increasing programme costs. Genetic, transgenic or symbiont-based sexing systems have been developed for a number of species [6, 18], but there are many species of interest for which these do not yet exist.

Tsetse flies present particular difficulties with sex separation. Characteristically, insects produce tens or hundreds of eggs per generation, so even if the sexes cannot be separated, pupae or adults of both sexes can be irradiated and released and still leave sufficient fertile insects to maintain the colony. In contrast, tsetse are adenoviviparous [22], producing one offspring every 10 days or 5–6 offspring in the female’s lifetime under insectary rearing conditions. This implies that female progeny need to be retained for colony maintenance and colony growth as much as possible. Furthermore, the eggs, embryos and larvae are developing in the uterus of the female and are therefore not accessible for genetic manipulation. Some progress has been made in this respect using paratransgenesis with the symbiont *Sodalis* [1, 37], but this method still needs refinement and has not yet been attempted for sex separation.

The principal method of sex separation of adult tsetse flies has been manual separation after chill immobilisation at 4 °C [17]. Adult male flies are morphologically readily distinguishable from females by the presence of the hypopygium, the modified 9th abdominal segment bearing the external genital structures of the male. However, separating tens of thousands female flies from the males is time consuming, e.g., for the SIT supported elimination programme on Unguja Island of Zanzibar [35], 21 technicians were employed to separate up to 110,000 males per week. For projects larger than this, manual separation is becoming cost inefficient and therefore attempts have been made to develop automatic sex separating systems. Early efforts to automate this by using image recognition software were unsuccessful as it was not possible to orient the adults accurately for the camera to reliably capture the hypopygium or hectors [19].

Tsetse biology does offer some opportunities, though. Previous studies showed variation in oxygen consumption and weight loss during pupal development. Spermatogenesis, a highly energetic process, takes place between day 11 and 20 and the spermatozoids appear mature between day 20 and 21 of pupal development [20, 21]. This can be observed in raised oxygen consumption and increased weight loss in males at this time. No practical method was, however, found to measure oxygen consumption in thousands of individuals separately. Attempts to use the weight loss to separate the pupae by floatation were ultimately unsuccessful as the saline floatation solutions proved harmful to the pupae. Another aspect that was studied was the capacitance of the developing adult cuticle within the pupa [33]. The hypopygium is folded underneath the 7th and 8th abdominal segments, so that in this region there is at least a triple thickness of cuticle. In principle, the capacitance of this thick lipid/protein structure can be measured and although the principle was demonstrated, no practical implementation for use in rearing facilities was found.

One aspect of tsetse biology, i.e., the protogyne of adult emergence from the pupae (most Diptera are protandrous) is conducive to sex separation of tsetse pupae. The pupal period of tsetse flies is around 30 days at 24 °C, varying a little with species, with females emerging on average two days before males. The first two days of emergence are almost exclusively female, and on days four and five almost exclusively male. Emergence on the third day is a male/female mix, but it supplies enough males for mating all the females in the laboratory colony (mating is often done at a 1:3 male:female ratio). If required, additional males can be added from day four. This process can be manipulated by adjusting the incubation temperature during the emergence period from 24 °C to 26.5 °C, which results in almost complete sex separation in some species [39, 40]. However, it has proven quite difficult in practice to maintain consistent separation, presumably due to issues of incubation temperature stability, and the system does not work well with some species due to a large overlap in female and male emergence.

Another issue that has driven the improvement of sexing systems is that the pharate males are on the point of emerging from the pupae when the female emergence finishes. The male pupae, therefore, must be chilled continuously to prevent emergence if they are to be irradiated and shipped to a distant release site. Prolonged chilling of the males can reduce their quality and competitiveness [9], so a method of separation that can be employed several days before female emergence starts would be desirable, as it would allow time for handling, irradiating and shipping without chilling. A system evaluated previously involved the placement of the newly deposited 3rd instar larvae in a saline solution. When deposited, the larvae crawl for about 100 min before pupariating, but when placed in saline this is reduced on average to 30 min for males and 50 min for females, but the overlap was still too great.

The difference in developmental time, oxygen consumption and weight loss between male and female tsetse flies suggests that there may be measurable differences in energy reserves in terms of chemical composition. Near infrared spectroscopy can detect various chemical bonds, including hydroxyl moieties that are energy rich. Single kernel near infrared spectroscopy (SKNIR) had been developed to assist with plant breeding, allowing the selection of desirable traits in individual grain kernels, including moisture and protein content, and hardness. During the development and use of this system, it became apparent that SKNIR has a wider applicability and could, for instance, detect insects inside the grains [3, 11] and even identify the genus of grain beetle [12], and age grading house flies [2, 28]. Investigations into the uses of SKNIR led to the assessment of whether tsetse can be sexed in the pupa. Immediately, clear differences in the short wavelength end of the spectrum (900–1100 nm) were visible with the absorption in this range rising abruptly six days before the first females emerge, followed by a matching rise in males two days later, corresponding to the difference in emergence date [10]. The spectra were, however, averages taken from many individual males and females, and although the system would work well one day, it would fail the next. At this stage, it was not clear what the spectral change represented although it was hypothesised to be changes in energy stores around the time of sperm maturation. When investigations switched from spectroscopy to infrared imaging, a breakthrough was made. It was observed that in the near infrared (850–1050 nm, the limit of standard silicon image sensor chips), the pupal shell becomes nearly transparent and the developing pupa and pharate adult could be observed inside the shell [23]. Sequential images and time lapse video showed that the change in the SKNIR spectrum of the female flies corresponded with the start of melanisation of the females. It also explained why the results from the SKNIR, which clearly registered the change, were unpredictable as the main part to melanise is the wings, which are only visible ventrally (the wings are wrapped under the venter and not visible dorsally, as illustrated in [23]). This paper describes the steps undertaken to capture and analyse images that would allow the sex of the tsetse fly to be determined between 6 and 4 days before first female emergence.

## Materials and methods

### Rearing and biological material

The *Glossina palpalis gambiensis* pupae used for the development and validation of the automated near infrared pupal sex sorter (NIRPSS) were obtained from a colony that was established in 1972 in Maisons-Alfort, France, using material collected in Burkina Faso [24]. The strain was transferred to the Centre International de Recherche-Développement sur l’Élevage en zone Subhumide (CIRDES), Burkina Faso, in 1975 and in 2009 material was provided to establish a colony at the Insect Pest Control Laboratory (IPCL) of the Joint FAO/IAEA Centre of Nuclear Techniques in Food and Agriculture, Seibersdorf, Austria [24]. The colony at the IPCL is maintained on defibrinated bovine blood (Svaman spol.s t.o., Majava, Slovakia) using an *in vitro* silicon membrane feeding system (under standardised environmental conditions 24–25 °C, 75 ± 5% R.H. and subdued/indirect illumination, 12 h light/12 h dark photoperiod) [14, 16].

Two synchronised pupae collections from the colony were made, at 09:00 in the mornings (18-hour larviposition period) and at 15:00 in the afternoons (6-hour period). After collection, pupae were incubated at 24.01 ± 0.04 °C and 76.65 ± 2.82% R.H.

### NIRPSS hardware

**Figure 1:**
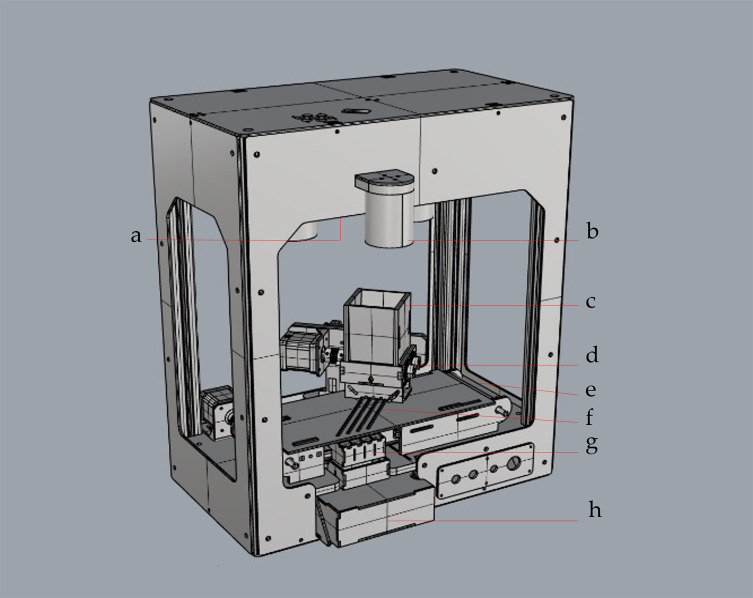
3D modelled view of the Near Infrared Pupal Sex Sorter (NIRPSS): (a) high speed camera with (b) NIR light-emitting diodes (LED), (c) 100 mL feeding container, (d) dosing device with parallel foam cylinders, (e) conveyor belt, (f) plate with four channels, (g) box with actuator flaps controlled by solenoids and (h) sorting drawers.

The NIRPSS is illustrated in Figure 1. Pupae from the 100 mL feeding container (Fig. 1a) pass through the dosing device that has a pair of parallel foam cylinders (Fig. 1d), positioned at the bottom of the container, turning slowly in opposite directions. Pupae are gently engulfed and fall through the slit in between the cylinders onto four channels in a polymethyl methacrylate (PMMA) plate (Fig. 1f). The pupae are continuously moving forward along the channels in a turning motion facilitated by a conveyor belt (Fig. 1e) underneath the plate. A high-speed camera without the IR blocking filter (IDS, model UI-3080CP-C-GL 2/3″ Rev2 2456x2054-86i/s-color CMOS-USB3) (Fig. 1a), placed above the plate, records frames of the pupae in all possible positions (dorsal, ventral and lateral) under infrared light created by four LEDs (ILS Intelligent LED Solutions, model ILH-IW04-85SL-SC211-WIR200, 850nm) (Fig. 1c) surrounding the camera. The camera is equipped with a global shutter, allowing acquisition of high resolution and high-speed images and avoiding distortion and blurring of the pupae in motion.

**Figure 2:**
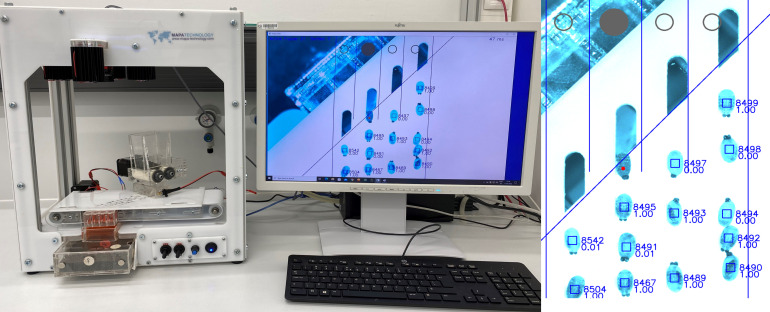
Near Infrared Pupal Sex Sorter (NIRPSS) connected to a computer during operation (left), and image of rows of pupae along the channels under NIR light with the melanisation index calculated in real time (right).

These images are analysed in real time by a software programme that calculates the degree of melanisation of each individual pupa, based on user defined parameters. At the end of the channel an actuator flap controlled by a solenoid (SparkFun Electronics, model Mini push-pull Solenoid – 5, ref: VROB-11015) (Fig. 1d) sorts the pupae into the sorting drawers (Fig. 1h) as either melanised or unmelanised (Figs. 1 and 2).

### NIRPSS software

The software for the sorter has been developed using the C^++^ language using Visual Studio ver. 15.7.5 as Integrated Drive Electronics and OpenCV 3.4.2 as Visual Library. It analyses the frames captured by the camera under near infrared light. Each pupa is segmented from the background applying a “threshold” filter with a parametric value to the image. Subsequently a filter “Canny” is applied. For the serialisation, blobs are identified using a filter to find contours and cleaned. Large blobs are considered an agglomeration of pupae and are divided according to their dimension. Each pupa is tracked individually across the collection of frames where it appears (±90 frames for each pupa) using predictive position algorithms that take into account the speed of the conveyor belt and the lapse between frames. The pupa has a different relative position (lateral, ventral and dorsal) in each frame, thus revealing a different melanisation pattern. In each collection of frames, a square of 35 × 35 pixels in the centre of the pupa is analysed to estimate the degree of melanisation. The colour of the pixels in the NIR single band spectrum varies between 0 (black) and 255 (white). After the user defines a threshold below which a pixel is considered melanised (parameter T1 or pixel intensity), the software calculates the proportion of melanised pixels in each frame square. If the proportion of melanised pixels is above a second user defined threshold (parameter T2), the pupa is considered melanised in that particular frame. If the percentage of melanised frames is above a third user defined threshold (parameter T3 or melanisation index), the pupa is classified as melanised and potential female. As the pupae reach the end of the channel, the software controls a flap actuated by a solenoid that sends the pupae to a drawer corresponding to its melanisation classification.

The interface is done through a configuration file where the user can define several parameters, such as T1, T2, T3, size of the rectangle of pixels to be analysed, conveyor belt speed, feeder speed, vibration pattern, serialisation parameter, among others.

### Experiment 1: Optimisation of melanisation parameters and optimal pupal sorting age

To determine the thresholds of the melanisation parameters (T1–T3) that result in the highest sex sorting accuracy and to ascertain the optimal pupal sorting age for a set maturation condition, images of individual *G. p. gambiensis* pupae were evaluated. Five batches of 60 pupae were recorded as individual pupae by the high-speed camera under NIR light, daily from day 19 to day 27 after larviposition. Pupae from different larviposition days were used and they were collected twice daily in order to synchronise the age of pupae in the same batch. The recorded images were analysed by the NIRPSS software, and the intensity of each pixel in the central square of the pupa for every frame was generated. Pupae were incubated until emergence and the sex of each fly recorded.

### Experiment 2: Evaluation of fly quality and sex sorting accuracy after pupae sorting

After the melanisation parameters and pupal sorting age were defined for optimal sex sorting accuracy, the potential effect of pupal sorting on emergence rate and flight propensity of the adult flies was assessed. *Glossina p. gambiensis* pupae that were incubated at a constant temperature (24.01 ± 0.04 °C) and relative humidity (76.65 ± 2.82%) for 24 days were sorted with the NIRPSS. As indicated above, pupae from different larviposition dates, collected twice daily, were used. For the first assessment, pupae were fed directly into the four channels without the dosing device (Fig. 1d). Pupae classified as unmelanised in this first sorting were sorted for a second and third time. After each sorting, 60 pupae from the unmelanised section of the sorting drawer were selected randomly for evaluation in a flight propensity quality control test. The experiment was replicated five times.

In a second assessment, a batch of pupae were again fed directly into the four channels and a second batch was first passed through the dosing device before entering the channels. The pupae from both batches that were sorted into the unmelanised section of the sorting drawers were then run three times through the NIRPSS. After the third sorting, 50 pupae from each batch sorted to the unmelanised section were selected for the flight propensity quality control test. The experiment was replicated six times. For the control groups, 60 pupae for assessment one and 50 pupae for assessment two were sub-sampled, before sorting, from the batches of pupae intended for sorting.

The flight ability of the adult flies was assessed using a standard flight quality control protocol, as described by [32]. Pupae from the control and sorted groups were placed in a flight quality control tube (10 cm high and Ø = 8.4 cm) and the emergence rate, flight propensity and sex of the emerged flies were determined. Before placing the pupae inside the flight tube, the inside of the tube was coated with unscented talcum powder to prevent the emerging flies crawling out of the flight tube. The pupae of the control and sorted groups were placed into separate petri dishes, which were placed inside the flight tubes inside a test cage (45 cm × 45 cm × 45 cm). Fly emergence as well as the numbers and sex of flies that had flown out of the flight tube and the flies that remain inside the tube were recorded daily. This continued until all flies had emerged.

### Experiment 3: Sex sorting accuracy for large batches of pupae

To assess the accuracy of the NIRPSS in sorting large numbers of pupae, *G. p. gambiensis* pupae were routinely sorted daily at the IPCL and Scientica (Slovak Academy of Sciences (SAS)) in Bratislava, Slovakia. The SAS colony was started from seed material from the IPCL, and the rearing conditions were similar to those at the IPCL. The pupae from the SAS colony were collected daily (synchronised pupae age under 24 h) and incubated under similar conditions as at the IPCL.

The daily number of pupae that were sorted varied at the insectaries, i.e., 3,000 to 13,000 pupae were sorted daily (large batch size) at the SAS and 250 to 2,500 pupae were sorted daily (small batch size) at the IPCL. At the SAS, pupae were sorted 22 or 23 days post-larviposition with an adjusted melanisation parameter for T3 between 10 and 30; at the IPCL, pupae were sorted 23, 24 or 25 days post-larviposition, using constant thresholds of the melanisation parameters. Pupae that were sorted into the unmelanised group were processed three times through the NIRPSS at both the IPCL and the SAS.

The sorted pupae were irradiated in air at ambient temperature with a dose of 110 Gy and shipped at ambient temperatures of 20–22 °C [15] to the Institut Sénégalais de Recherches Agricoles/Laboratoire National de l’Élevage et de Recherches Vétérinaires (ISRA/LNERV) in Dakar, Senegal to be used in the SIT campaign that was implemented in the Niayes around Dakar. Each week ISRA/LNERV would receive two shipments containing pupae sorted at the SAS and the IPCL. The daily sorted pupae for each insectary were combined and subsampled, 100 pupae from the SAS and 50 pupae from the IPCL, were used for flight quality control assessment [35]. The recovery of male (RM) and loss of females (LF) of each shipment batch of each insectary was determined by estimating the following probabilities:

$$\mathrm{RM}=P(\mathrm{unmelanised}|\mathrm{male})=\frac{P(\mathrm{unmelanised})P(\mathrm{male}|\mathrm{unmelanised})}{P(\mathrm{male})},$$

$$\mathrm{LF}=P(\mathrm{unmelanised}|\mathrm{female})=\frac{P(\mathrm{unmelanised})P(\mathrm{female}|\mathrm{unmelanised})}{P(\mathrm{female})}.$$

*P*(unmelanised) was estimated as the ratio between unmelanised pupae and the size of the batch. *P*(male|unmelanised) and *P*(female|unmelanised) were estimated as the ratio between the number of males (females) emerged in the subsample and the total number of emerged pupae in the subsample. An equal proportion of males and females in all batches was also assumed (*P*(male) = *P*(female) = 0.5).

### Statistical analysis

Data from experiment 1 were analysed with R statistics package [29] with libraries ggplot2 [38] and pROC [30]. A ROC curve for male and female recovery rates was computed for multiple combinations of T1 and T2 parameters and optimal T3 threshold was calculated for each configuration.

The data from experiments 2 and 3 were analysed with RStudio [31] using R software version 4.1.2. with the packages: ggplot2 [38] and ime4 [4]. A generalised linear mixed-effect model fitted by maximum likelihood with a “binomial (logit)” family was used to analyse the flight propensity data, the emergence rate and proportion of flyers. For the flight propensity data, the emergence rate and rate of flyers were the response variables, the control and sorted groups were used as fixed effects, and replicates were used as random effect. For the sorting accuracy of large batches, a linear model was used, male recovery and loss of females were the response variables, the sorting location (sorting batch size) was used as fixed effect, and shipment lot was used as random effect.

## Results

### Experiment 1: Evaluation of the sex sorting accuracy

**Figure 3:**
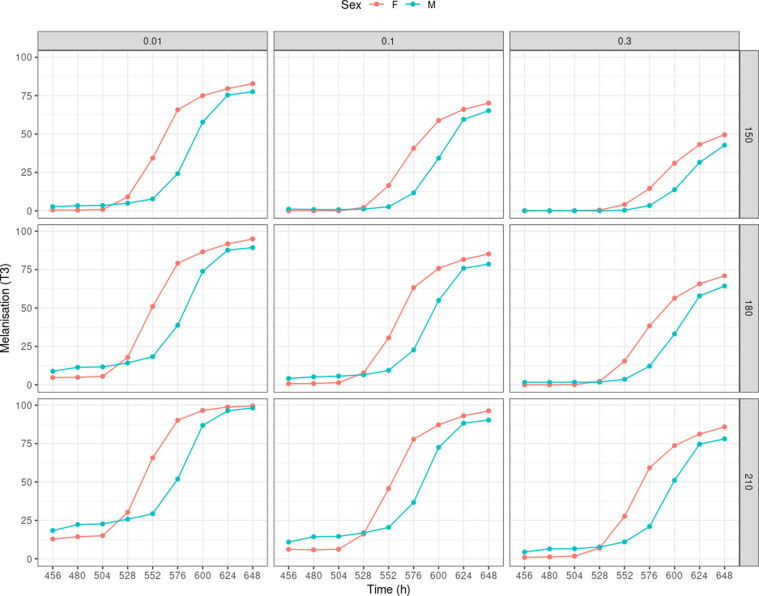
Mean values of the melanisation index (T3) for several combinations of T1 (rows) and T2 (columns) at different pupae maturation intervals. Data collected from male and female pupae of five batches of 60 pupae combined.

**Figure 4:**
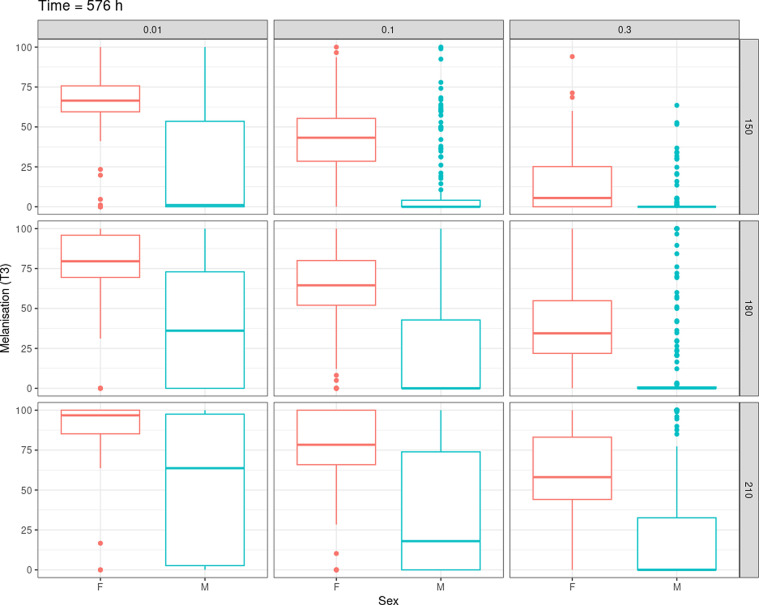
Boxplots of the melanisation index (T3) for different combinations of T1 (rows) and T2 (columns) on day 24 post larviposition (576 h).

**Figure 5:**
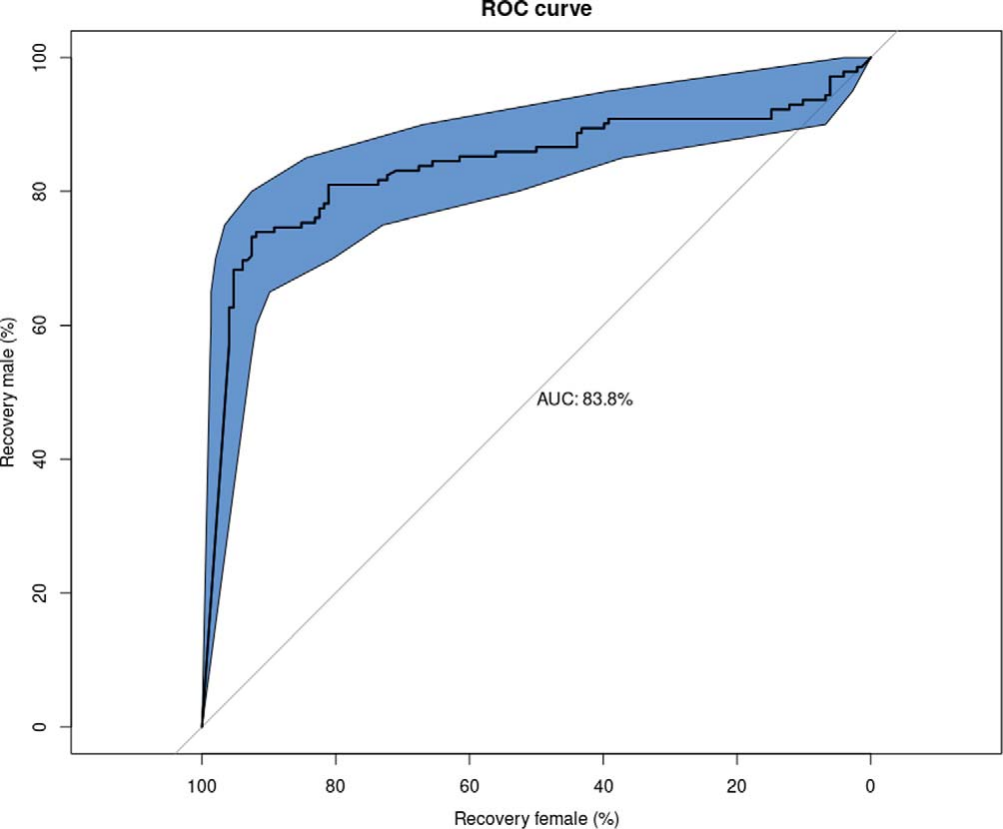
ROC curve for T1 = 140, T2 = 0.01. Blue shaded area depicts the envelope of the confidence intervals (95%) for male recovery rate at a given female recovery rate.

The melanisation index (T3) was calculated for both male and female pupae for several combinations of the pixel intensity (parameter T1) and rate of melanised pixels in the central square (parameter T2) at different time intervals (from 456 h post-larviposition to 648 h post-larviposition every 24 h). The results for the five batches of 60 pupae combined (Fig. 3) indicate that the sorting carried out on day 24 post-larviposition (576 h) gives the lowest overlap between males and females for all combinations of T1 and T2. The distribution of the melanisation index (T3) is highly dependent on the user-defined thresholds T1 and T2 (Fig. 4). In order to find combinations of parameters with optimal sorting power, screening on T1, T2 space was carried out. For each (T1, T2) duple, a ROC curve was calculated (Fig. 5). Confidence intervals (95%) for male and female recovery rates were computed with bootstrap replicates. Configurations with at least a simultaneous 60% male and 90% female recovery rate were selected to calculate the T3 threshold. Due to the interplay between T1, T2 and T3, there is not a single optimal set of parameters, but multiple combinations achieved the desired sex sorting goals.

### Experiment 2: Evaluation of fly quality and sex sorting accuracy after pupae sorting

**Figure 6:**
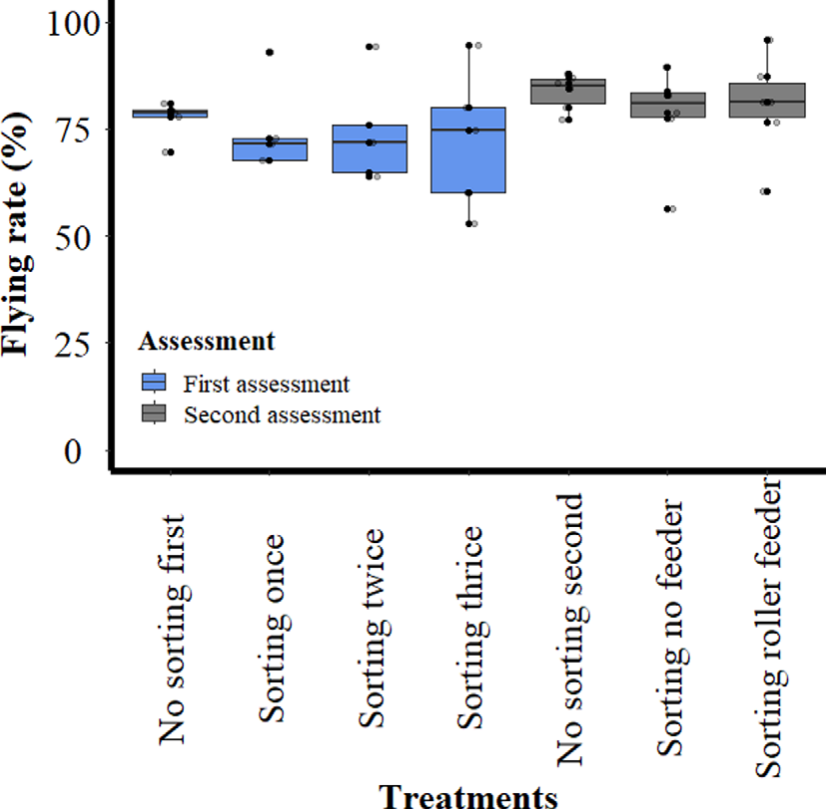
Percentage of flyers that escaped the flight tube after *Glossina palpalis gambiensis* pupae were sorted with the Near Infrared Pupal Sex Sorter (NIRPSS). Each box shows the group median separating the 25th and 75th quartiles, bars indicate maximum and minimum values, circles indicate the outliers.

For the first assessment, 5,694 pupae were sorted during five replications. During the first sorting, a mean of 1,138.8 ± 191.13 pupae were sorted in each replicate. Thereafter only the pupae that were classed as unmelanised were sorted a second (mean 358.6 ± 138.16) and a third time (mean 271.0 ± 133.60). The mean unmelanised ratio, number of pupae classed as unmelanised divided by the total number of pupae sorted, for the first sorting (36.08 ± 7.47%) was significantly different (*p* < 0.006) to that of the second and third sorting. The mean unmelanised ratio for the second- and third-time sorting did not change significantly (*p* = 0.510), with means of 36.08 ± 7.47%, 33.67 ± 7.22%, and 33.11 ± 7.11%, respectively. The mean emergence rate for the unsorted control pupae and pupae sorted once, twice and three times were 96.99 ± 3.97%, 94.33 ± 2.24%, 93.92 ± 4.09% and 92.61 ± 1.41%, respectively. The emergence rate for pupae that were sorted three times differed significantly (*p* = 0.039) from that of unsorted pupae. No other significant differences were observed for the other sorting groups. The mean male recovery was similar in the first (61.18 ± 9.99%), second (59.44 ± 10.72%) and third sorting (59.75 ± 9.90%). The change in the mean rate of flyers (Fig. 6) from 77.26 ± 4.50% for the control to 74.56 ± 10.53%, 74.16 ± 12.29% and 72.36 ± 16.53% after one, two and three sortings, respectively was not significant (*p* > 0.148).

**Figure 7:**
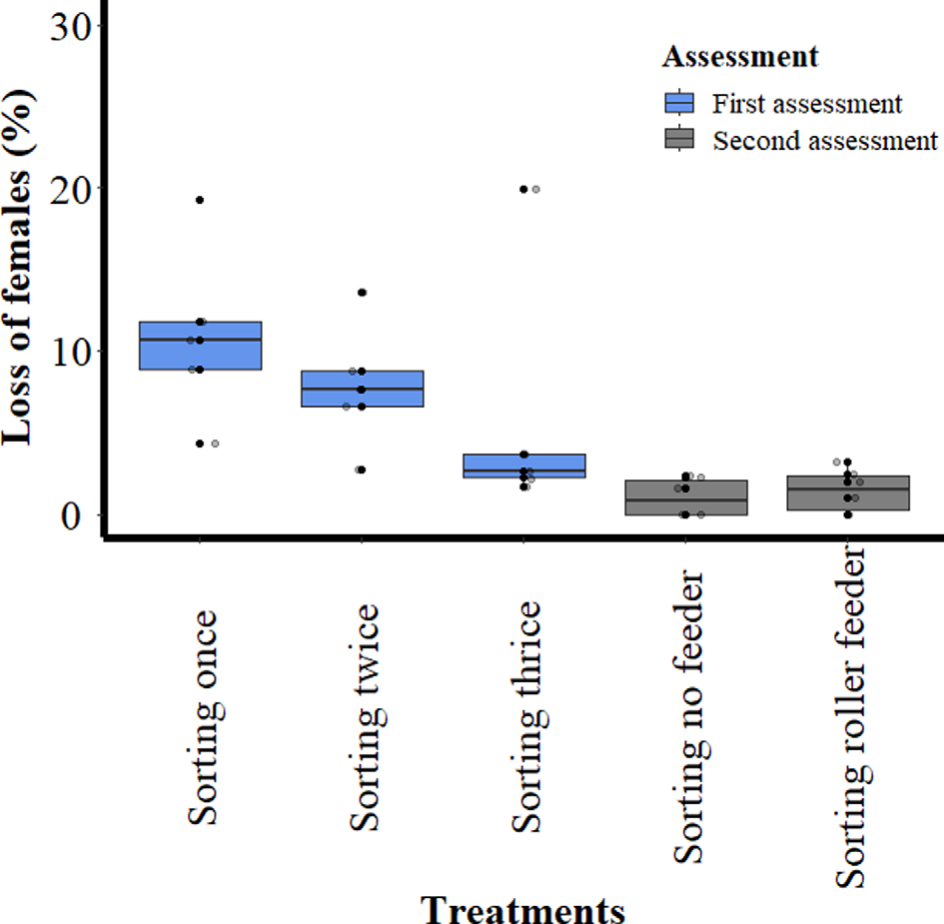
Percentage loss of females after *Glossina palpalis gambiensis* pupae were sorted with the Near Infrared Pupal Sex Sorter (NIRPSS). Each box shows the group median separating the 25th and 75th quartiles, bars indicate maximum and minimum values, circles indicate the outliers.

The mean loss of females (Fig. 7) after one sorting was 10.98 ± 5.45% and it decreased to 7.86 ± 3.96% and 6.01 ± 7.81% after the second and third sorting. There was no significant difference between the pupae that were sorted once compared to the pupae that were sorted twice; however, there was a significant difference in the loss of females between the pupae that were sorted once and those sorted three times (*p* = 0.014).

In the second assessment, a total of 3,699 (mean 616.5 ± 124.33) pupae were loaded directly into the four channels (without feeder) and a second group of 3,561 pupae (mean 593.5 ± 123.05) were passed through the dosing device (with roller feeder) into the four channels during six replicates. The mean unmelanised ratio for the pupae that were sorted without a feeder was 21.70 ± 5.72% and for the pupae sorted with the roller feeder 24.30 ± 2.94%.

The mean time (seconds) to sort (unmelanised classed pupae are processed through the NIRPSS three times) one pupa without the feeder (0.53 ± 0.06 s) was marginally shorter than with the feeder (0.61 ± 0.11 s). The mean emergence rate for the control and sorted pupae groups (without or with roller feeder) were 96.00 ± 4.20%, 96.00 ± 1.79%, and 94.00 ± 4.20%, respectively. In contrast to assessment one, there was no significant differences for the emergence rates between the control and the sorting groups or between the sorting groups.

The mean male recovery was lower for the pupae sorted without than with the roller feeder, i.e., 42.36 ± 10.68% and 45.80 ± 6.48%, respectively. The mean rate of flyers (Fig. 6) of the control group was 83.70 ± 4.27% and not significantly different to that of the flies emerging from the pupae that were sorted without (78.13 ± 11.53%) or with (80.47 ± 11.86%) the roller feeder. The mean loss of females (Fig. 7) was lower than recorded in the first assessment as it was 1.03 ± 1.16 for the pupae that were sorted without the feeder and 1.43 ± 1.31 for pupae that were sorted with the roller feeder. However, there was no significant difference (*p* > 0.147) in the loss of females between the pupae that were sorted without or with the roller feeder.

### Experiment 3: Sex sorting accuracy for large batches of pupae

A total number of 1,329,074 (251 sorting batches) and 229,703 (280 sorting batches) pupae were sorted at the SAS and the IPCL, respectively from March 2021 to January 2022. The batch sizes varied at the SAS (large batch) from 3,380 to 12,627 with a mean batch size of 6 559 ± 1 633.90, and at the IPCL (small batch) from 241 to 2 554 with a mean of 829.94 ± 394.20. The mean unmelanised ratio for the pupae sorted at the SAS and the IPCL was 33.75 ± 1.27 and 24.13 ± 6.71, respectively.

**Figure 8:**
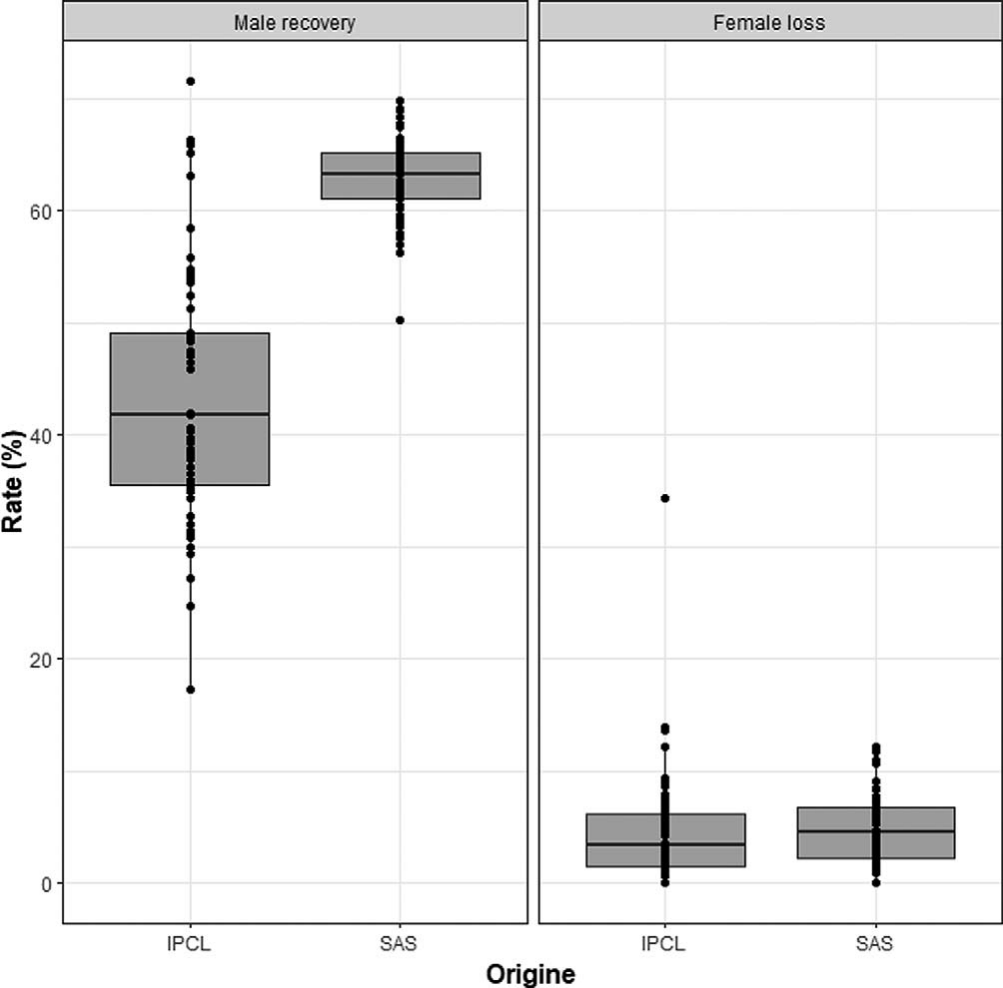
Male recovery and loss of females for small and large batches of *Glossina palpalis gambiensis* pupae that were sorted with the Near Infrared Pupal Sex Sorter (NIRPSS) at the Insect Pest Control Laboratory (IPCL) and the Scientica (SAS). Each box shows the group median separating the 25th and 75th quartiles, bars indicate maximum and minimum values, circles indicate the outliers.

The sorted pupae were irradiated and shipped to ISRA/LNERV in 71 shipments. The mean male recovery was 62.82 ± 3.61 for the pupae that were sorted from the large batches (SAS) and 43.59 ± 11.36 for the small batches (IPCL) and the mean loss of females was 4.69 ± 3.02 (SAS) and 4.67 ± 5.24 (IPCL) (Fig. 8). The male recovery for the pupae that were sorted from the large batches at SAS was significantly higher (*p* < 2e–16) than for the pupae sorted from the small batches at the IPCL. There was, however, no significant difference in the loss of females for the pupae that were sorted from the large or small batches.

## Discussion

The feasibility of the application of SIT for the sustainable elimination of selected populations of tsetse flies has been demonstrated on Unguja Island of Zanzibar and in the Niayes of Senegal. In addition, the cost-effectiveness of the application of SIT within an AW-IPM approach to eliminate an isolated tsetse population was amply demonstrated in the programme in the Niayes [7]. Despite this, SIT for tsetse elimination is not widely used, one of the reasons being the difficulty in scaling up and maintaining large colonies of tsetse flies to obtain the required number of sterile males. The NIRPSS described here can potentially simplify tsetse mass rearing procedures and reduce the sterile male production costs.

The evaluation of sex sorting accuracy indicates that the optimal age for sorting *G. p. gambiensis* pupae is on day 24 post larviposition, in pupae maintained at a constant temperature 24.01 ± 0.04 °C and 76.65 ± 2.82% R.H. Under these conditions, melanisation of some distinctive organs occurs in most female pupae, whereas male pupae remain largely unmelanised, except for the eyes. The shell of tsetse pupae is nearly transparent to near infrared light, allowing observation of the melanised organs of the pharate fly. The melanisation pattern of the pharate fly inside the shell is not homogeneous and its analysis depends on the relative position of the pupa. To overcome this difficulty, pupae are rotated along their longitudinal axis under a high-speed camera and a melanisation index is calculated by the software after analysing the collection of frames of each pupa in real time. The melanisation index determining whether a pupa is considered melanised results from an algorithm computing the rate of dark pixels in the central part of the pupa across the collection of frames. The set of thresholds for this index that optimises the recovery of male pupae, while maintaining the loss of females below an acceptable level of 10%, has also been determined. This complex algorithm was designed to allow the proper identification of the pupae melanisation status, even if a pupa is only partly melanised and its melanised organs are only visible in certain orientations.

The evaluation of the effect of NIRPSS on the quality of the males showed that multiple sorting had no significant negative impact on the emergence rate of pupae. No significant wing damage could be found and there was no negative effect on the male’s ability to fly after the sorting with or without the roller feeder. The loss of females was significantly reduced when the pupae classed as unmelanised were sorted two more times after the initial sorting. It is thus recommended that this resorting should be included in the sorting protocol.

Manual sex sorting of immobilised chilled tsetse adults is a labour-intensive and a time-consuming activity in mass-rearing facilities. Chilling and manual separation of sexes takes 23% of the time invested in tsetse mass rearing [19]. In contrast, the NIRPSS can sort up to 36 pupae per minute (> 2,100 per hour), including resorting (twice) of the unmelanised classed pupae. This yield is sufficiently high to sort in four hours per day pupae production of a tsetse colony of 144,000 females with a fecundity of 0.6 pupae per female per 10 days, an average value for most tsetse species [14]. Furthermore, one technician can operate two NIRPSS simultaneously, as they require low attendance.

In the present study, the sorting potential of the NIRPSS was evaluated in two relatively small colonies of *G. p. gambiensis*. Larger colonies consisting of up to 500,000 females as maintained in Burkina Faso or Ethiopia, considering the four hours of working time per machine per day, may require that several NIRPSS will be needed for daily sorting of pupae. Despite the initial relatively higher input cost, compared to that of the chiller needed for manual sorting, the introduction of NIRPSS in the long term can be cost effective based on the assumption that with the NIRPSS one technician can sort approximately 4,200 pupae per hour compared to manual sorting where one experienced technician can sort approximately 1,000 flies per hour using a chiller and human error will be minimised. More important advantages of the NIRPSS, though, are the avoidance of chill damage to the flies during manual sorting and the simplification of shipping pupae that are not on the point of emergence. Despite the obvious advantages described, it needs to be mentioned that cost-effectiveness of the introduction of the NIRPSS will depend on the infrastructure, labour costs and manpower availability in each location.

In a recent AW-IPM against tsetse flies, sterile male pupae were produced in mass-rearing facilities that are not located in the vicinity of the release areas, thus requiring the shipment of sterile male pupae over long distances [32, 36]. In the case of the project for the elimination of *G. p. gambiensis* in the Senegal Niayes, sterile male pupae were produced in facilities located in Burkina Faso, Slovakia and Austria, and then shipped to Senegal with a minimum transportation time of three days [9]. Most of the sex separation of pupae at tsetse laboratories are based on differences in emergence time of females and males [9, 27]. After the majority of the females and around a third of males has emerged within the first three days of emergence, the remaining pupae at the point of emergence are mostly males and can be irradiated and shipped. Although emergence can be halted by maintaining the pupa below 12 °C [5], mechanical disturbance and temperatures above this threshold result in the initiation of emergence [27]. As irradiated pupae are at the point of emergence, they must be immediately packed in insulated boxes with sufficient phase-change material packs to maintain the temperature inside the box between 8 °C and 10 °C for the duration of the shipment, usually 72 h [27]. Longer transport times caused by customs or flight delays lead to the emergence of adults during shipment resulting in flies with deformed or damaged wings and loss of operational sterile males for the programme. In more than 55% of shipments, fly emergence was observed during transport [27]. Also, because pupae shipments for the programme in Senegal were only done twice per week, sterile male pupae must be kept at 10 °C in the insectaries for several days before shipment. The duration of these chilling conditions has a negative impact on the performance of the sterile males in terms of flight propensity and survival [25, 26]. The NIRPSS has also improved the logistics of long-distance shipments of sterile male pupae from the mass-rearing facility to the field release area. Since pupae are sorted 5 days before the emergence of the first females and 7–8 days before the emergence of the males, sterile pupae do not need to be kept at low temperatures before and during the transboundary shipments and can be kept and shipped at 20 °C. The fact that all pupae shipped from Slovakia (SAS) and Austria (IPCL), since 2020 were separated with the NIRPSS and shipped under unchilled conditions demonstrates the feasibility of the method. In evaluating these results, it needs to be taken into account that variation in the sorting protocol, as found to be practical in the different laboratories, may influence the results. At the SAS, between 3,000 and 13,000 pupae were collected and sorted daily when they were between 22 or 23 days old and the melanisation parameter T3 was adjusted for each sorting event to ensure an unmelanised ratio as close to 30% as possible. This combination of sorting the pupae between a smaller age range and the adjusting of the melanisation parameter for each sorting event resulted in a higher mean male recovery than that observed at the IPCL. The mean loss of females was, however, the same in both insectaries. Large numbers of pupae, up to 12,627 per batch, can be sorted successfully with a mean male retention of 53.10% ± 13.12% and mean female contamination of 4.67 ± 4.26%. While tsetse pupae sex sorting with NIRPSS has some clear advantages, it requires standardisation in tsetse insectaries: pupae collection and sex sorting must be done daily, following standard operating procedures. Although the rearing conditions as well as the incubation condition at the two insectaries were similar, a difference in the appropriate age needed for accurate sorting was observed. As the temperature during pupae maturation is a critical factor, pupae incubation should be done in rooms with reliable control of environmental conditions, preferably in incubators with not more than ±0.1 °C variation in temperature. The sorting accuracy can be further improved by refining the appropriate sorting age (between 23–25 days for *G. p. gambiensis* pupae) as this is temperature-dependent and the sorting age point can vary slightly with different incubation conditions. Furthermore, improvements in sorting accuracy can be achieved by customising the values of T1, T2 and T3 for each pupae sorting batch by sorting a small number of pupae (100). It needs to be stressed that the current study involved a single species, i.e., *G. p. gambiensis* and that the effectiveness for other tsetse species still needs to be determined and evaluated. The wider use of the NIRPSS, and the inclusion of more species, may eventually lead to an improvement in efficacy.

## Conclusions

We demonstrated that pupae of *G. p. gambiensis* can be sex-sorted by assessing the melanisation status of 24-day old pupae through image analysis of pupae frames recorded under near infrared light. This method can potentially significantly reduce the workload at tsetse insectaries by simplifying rearing procedures, resulting in lower production costs. Furthermore, it allows the shipment of non-mature sterile male pupae from mass-rearing centres to field programmes, avoiding the need to maintain low temperatures during transportation of pupae. It is expected that the sex sorting procedure with NIRPSS can be adapted to other tsetse species and adjusted to specific rearing conditions, as found in different laboratories.

## Conflicts of interest

The authors declare that they have no conflicts of interest.
